# Concurrent Conditional Clustering of Multiple Networks: COCONETS

**DOI:** 10.1371/journal.pone.0103637

**Published:** 2014-08-08

**Authors:** Sabrina Kleessen, Sebastian Klie, Zoran Nikoloski

**Affiliations:** 1 Systems Biology and Mathematical Modeling Group, Max Planck Institute of Molecular Plant Physiology, Potsdam-Golm, Germany; 2 Genes and Small Molecules Group, Max Planck Institute of Molecular Plant Physiology, Potsdam-Golm, Germany; University of Turin, Italy

## Abstract

The accumulation of high-throughput data from different experiments has facilitated the extraction of condition-specific networks over the same set of biological entities. Comparing and contrasting of such multiple biological networks is in the center of differential network biology, aiming at determining general and condition-specific responses captured in the network structure (*i.e.*, included associations between the network components). We provide a novel way for comparison of multiple networks based on determining network clustering (*i.e.*, partition into communities) which is optimal across the set of networks with respect to a given cluster quality measure. To this end, we formulate the optimization-based problem of concurrent conditional clustering of multiple networks, termed COCONETS, based on the modularity. The solution to this problem is a clustering which depends on all considered networks and pinpoints their preserved substructures. We present theoretical results for special classes of networks to demonstrate the implications of conditionality captured by the COCONETS formulation. As the problem can be shown to be intractable, we extend an existing efficient greedy heuristic and applied it to determine concurrent conditional clusters on coexpression networks extracted from publically available time-resolved transcriptomics data of *Escherichia coli* under five stresses as well as on metabolite correlation networks from metabolomics data set from *Arabidopsis thaliana* exposed to eight environmental conditions. We demonstrate that the investigation of the differences between the clustering based on all networks with that obtained from a subset of networks can be used to quantify the specificity of biological responses. While a comparison of the *Escherichia coli* coexpression networks based on seminal properties does not pinpoint biologically relevant differences, the common network substructures extracted by COCONETS are supported by existing experimental evidence. Therefore, the comparison of multiple networks based on concurrent conditional clustering offers a novel venue for detection and investigation of preserved network substructures.

## Introduction

Network representations of biochemical system, extracted from high-throughput data, accumulated knowledge, or combination thereof, have become prominent in modern systems biology [1]. Biological networks consider the interconnections between components (*e.g.*, genes, proteins, metabolites), and may capture the involvement in biochemical reactions, physical proximity (*e.g.*, on the DNA), or other types of interactions. Experimental evidence has indicated that biochemical networks are plastic in the sense that their functionality is altered under different internal and external perturbations (*e.g.*, due to genetic manipulations and change in condition, respectively), manifested in the resulting cellular behaviors [2]. Therefore, to reveal the changes in biochemical networks underlying different perturbations, it is necessary to develop methods for comparison of multiple biological networks. This type of analysis is a part of *differential network biology*, comprising computational methods for comparison of biochemical networks based on: (1) alignment, in the case of networks over different but evolutionary related components, or (2) (weighted) difference, in the case of networks over the same set of components [3].

**Figure 1:**
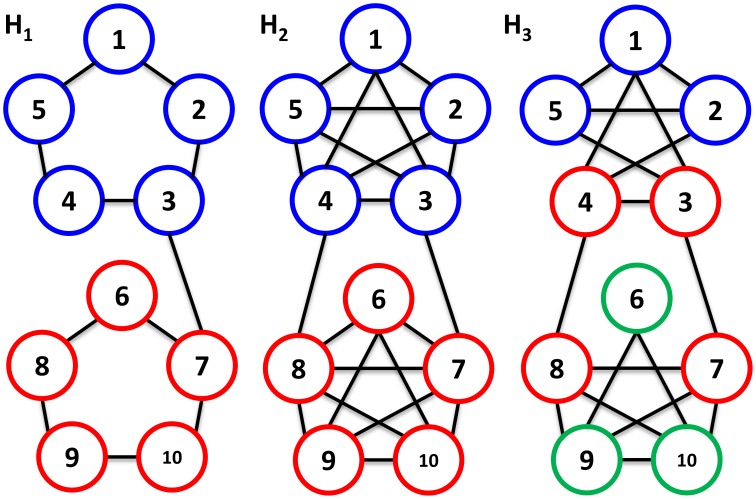
Illustration of network comparison based on community structure. Shown are three networks, 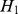, 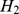, and 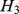. Nodes belonging to the same community in each network are marked by the same color. Networks 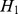 and 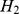 differ in 11 edges, while networks 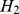 and 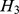 do not share 4 edges. Nevertheless, the community structures between 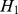 and 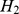 are equivalent, while this is not the case for the community structures in 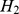 and 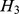.

Here we focus on comparison of multiple networks over the same set of components, and explore a conceptually different way for multiple network comparison based on differences between their clusteredness, *i.e.*, their network community structure. The idea is based on the observation that classical network operations, *e.g.*, intersection and difference, stress the absolute (dis)agreement between the edge-sets in two compared networks, although the community structure of the two networks may not be drastically altered with removal and/or addition of subset of edges. A motivating example includes three networks, 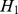, 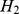, and 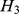: although 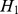 and 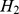 share fewer edges in comparison to 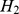 and 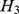, the given community structures of 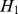 and 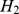 are closer than the community structures of 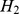 and 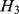 (Figure 1).

Comparison of clusterings has received considerable attention in information theory, data mining, and statistics, and has resulted in several indices, including: the Rand index, variation of information, and the cluster homogeneity index, which can be readily employed [4]. Moreover, in combination with biochemical knowledge, structured in a form of ontologies, the resulting network clusters, popularly referred to as network communities, can be analyzed for enrichment of concepts used in the annotation of the interconnected biochemical components [5]. Therefore, our novel approach for multiple network comparison relies on the established observation that cellular networks encompass interacting structural modules of different functional roles [6]. Moreover, in the case of network extracted from data, such an approach allows for unsupervised investigations of high-throughput data sets [7], [8].

Our approach relies on first determining a clustering of components which is optimal *across* a set of networks with respect to a given cluster quality measure. The resulting clustering will depend on all networks and can, thus, pinpoint their preserved substructures. Furthermore, this clustering can be readily compared with the optimal (with respect to the used cluster quality measure) from one of the investigated networks to determine the specificity of structural differences, previously not undertaken in other studies. The optimization-based formulation for the problem of clustering multiple networks is based on the concept of modularity [9], a widely-used cluster quality measure. We term the proposed formulation *Concurrent Conditional clustering of multiple Networks* or briefly COCONETS. Given a set of networks over the same set of components (*i.e.*, nodes), COCONETS involves determining a clustering 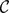 such that the nodes in the same cluster of 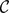 are more similar to each other than those in different clusters of 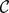 *over all* networks. The *concurrent* clustering of multiple networks is in fact also implicitly *conditioned* on the topology of each of the considered networks. More precisely, COCONETS encompasses clustering of multiple network with respect to a given cluster quality index—here given by the modularity. Therefore, like the problem of clustering single networks based on maximizing the modularity, our approach also has the advantage that it does not require specification of the number of clusters as an input parameter.

As COCONETS is NP-hard, we investigate a greedy heuristics for obtaining a solution, termed CoCo clustering. Moreover, we obtain theoretical results for the optimal CoCo clustering for a combination of complete graphs and cycles, which are used to evaluate the heuristic used in the subsequent empirical analysis. We show that the proposed greedy heuristics, applied on (time-resolved) transcriptomics data sets from *Escherichia coli* under five different conditions, can be effectively employed to identify biological processes and molecular functions which are preserved across the investigated conditions and are in line with the existing biological knowledge. Moreover, the comparison of CoCo clusterings from a subset of networks allows determining specificity of transcriptional response of *E. coli* to the investigated stresses. In addition, we applied COCONETS to a publically available metabolomics data set including the time-resolved courses of 101 metabolites from *Arabidopsis thaliana* exposed to eight environmental conditions differing in light intensity and/or temperature. The investigation of clusterings from subsets of networks as well as from all networks show to identify metabolites whose behavior is similarly affected due to the different perturbations.

## Approach

### Preliminaries on networks

Throughout the paper, we will rely on the notation used in [10], [11]. We assume that there are 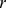, 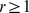, undirected networks (graphs), 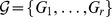, over the same set of nodes, *i.e.*, 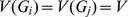, 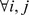, 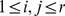. Moreover, let 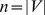 denote the number of nodes and 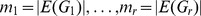 be the numbers of edges in each of the 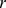 networks in 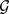. Furthermore, let 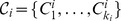 represent a partition of 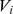, 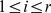, *i.e.*, 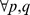, 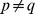, 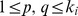, 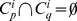 and 
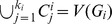
. We call 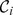 a *clustering* of 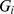, and 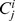, with 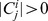, 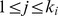 denote the *clusters* of 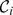. We denote the set of all possible clusterings of 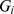 by 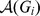, 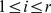, and the set of all possible clusterings over all 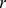 graphs by 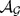. Note that each cluster 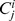, 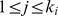, of 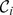 induces a subnetwork in 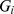, *i.e.*, 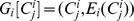, where 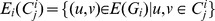. Then 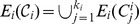 is the set of *intracluster edges*, and 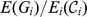 is the set of *intercluster edges* in 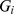.

### Modularity

Modularity is a cluster quality index which has found numerous applications in partitioning of networks into communities (*i.e.*, clusters) without *a priori* specifying the number of clusters. Given a network 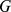 and a clustering 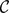 of its nodes, the modularity of the clustering 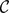 given a network 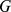, denoted by 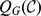, is defined as follows [9]:
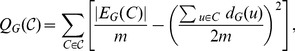
(1)where 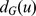 denotes the degree of node 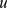 in 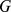 and 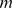 is the number of edges in 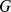.

The Modularity problem refers to that of determining a clustering 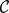 of maximum modularity in 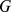. It has been shown that the Modularity problem is strongly NP-hard, and that the 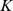-Modularity problem, whereby one is to determine the clustering into 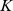 clusters of maximum modularity, is also strongly NP-hard even when 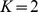 [11]. Multiple heuristics have been proposed to determine a clustering which approximates the optimal value of modularity for a given graph 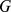. These are based on different clustering approaches, including: agglomerative clustering [12], [13] and spectral division [14], [15], as well some techniques from optimization, including: simulated annealing [16], [17] and extremal optimization [18]. It has also been shown that there exists a class of graphs on which the agglomerative clustering heuristic, proposed in [12], does not have a finite approximation factor [11].

### Enrichment analysis and adjusted Rand index for cluster comparison

Given two clusterings, 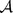 and 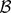, over the same set of 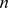 components, the Rand index is the proportion of pairs which agree with respect to their placement in same or different clusters in 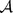 and 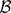 [19]. Thus, it quantifies the similarity between two clusterings. Since the expected value for the Rand index is non-zero and may inflate the similarity which could arise by chance, the original formulation of the Rand index has been modified, yielding the adjusted index 
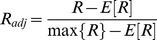
, where 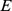 denotes the expected value under the hypergeometric null model. Values of 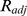 closer to zero, indicate that the value for the original Rand index is close to that expected by chance; hence, the compared clusterings are deemed non-significantly similar. A value closer to 1 indicates highly similar clusterings. For a given clustering and a set of annotation terms from an ontology, the hypergeometric distribution is employed to determine the set of terms enriched in each of the clusters at a chosen significance level.

### Approach formulation

In this section, we provide an optimization-based formulation for COCONETS.

**Definition 1**. *A clustering* 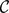 *of* 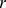 *networks* 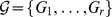 *over the same set of nodes is said to be optimal concurrent conditional clustering if it maximizes* 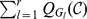.

By restriction to the case when 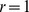, *i.e.*, there is only one network to be clustered, the COCONETS problem is NP-hard due to the NP-hardness of Modularity [11]. The optimal clusterings per Definition 1 can be divided into those which are also optimal in each of the networks and those which are suboptimal in at least one of the networks. In fact, the conditional clustering of networks stems from considering those clusterings which are suboptimal in at least one of the networks, but satisfy the optimality of the clustering across all networks, as required by Definition 1.

**Figure 2:**
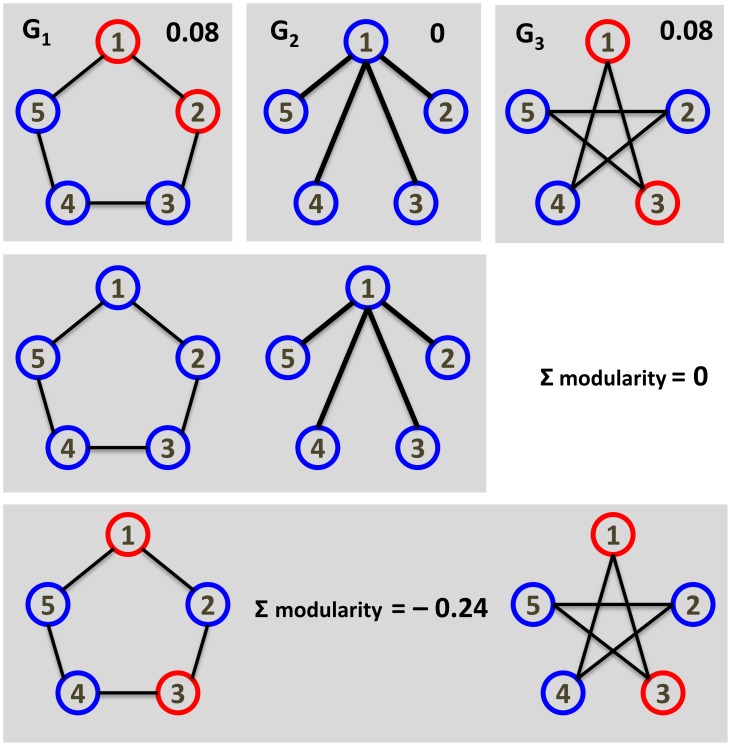
Illustration of COCONETS. Given are three networks in the top row. The clusters in the optimal clustering for each network are marked in different colors (red and blue). The optimal concurrent conditional clustering of the two networks given in the middle row is of value 0 and is suboptimal for the network to the left. The optimal clusterings for any of the two networks shown in the bottom row are suboptimal concurrent conditional clustering; the shown clustering yields a value of −0.24, while the optimal clustering is of value 0, whereby all nodes form a single cluster (not shown).

For instance, consider the case of three networks: 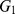, a cycle on 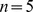 nodes, 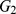, a star on the same number of nodes with the node labeled 1 as its center, and 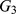, a cycle with permuted labels. It can readily be checked that the clustering of maximum modularity for 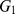 contains two clusters: one containing two neighboring nodes, and the other the rest of the nodes, yielding a value of 0.08. The same argument holds for 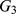 (accounting for the permutation). The optimal clustering for 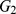 includes all nodes, yielding a value of 0 for the modularity. The clustering maximizing the sum of modularities for 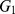 and 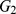 coincides with the clustering for 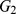, yielding also a value of 0; this clustering is suboptimal for 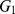, but optimal in 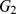. The clustering maximizing the sum of modularities for 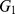 and 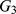 includes all nodes, resulting in a value of 0; moreover, it is suboptimal in both 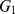 and 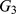, exhibiting value greater than 0 for the respective optimal clusterings (Figure 2).

#### COCONETS *vs.* other clustering alternatives

While the concurrent conditional clustering of multiple networks has not received much research attention, there have been numerous solutions proposed for the similar but more specific problem variants, including that of *consensus clustering* and *simultaneous clustering* of different data sets (*i.e.*, not necessarily networks). COCONETS is more general than the *consensus clustering* [20], whereby one determines the clustering (partition) 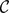 with respect to an objective function operating on a given set of clusterings 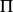 as its domain. For instance, clustering of clusterings based on the co-association value [21] and the notion of median partition [22] have been investigated as objectives. The generality of COCONETS follows from the fact that 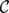 is to be determined from the set of all possible partitions 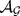 over the set of networks 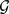, and not only with respect to the clusterings in the given set 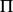. A similar problem has been analyzed with the idea of determining common temporal gene expression profiles across experimental conditions [23], whereby clustering based on polynomial models is first extracted from each condition and the resulting collection of clusterings is subjected to the same clustering method but only in order to merge them (*i.e.*, without considering concurrency in the process of clustering).

In addition, COCONETS differs from *simultaneous clustering of multiple networks*, whereby one is to find a clustering 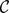 which satisfies a set of *a priori* given constraints, seen as parameters, which may be network-specific. For instance, given a set 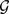 of 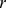 networks, the most recently proposed JointCluster approach [24] relies on the attractive properties of spectral clustering and relations to the well-established cluster quality index called conductance [25]. JointCluster consists of finding a clustering 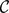 where the conductance of each cluster 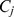, 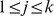 is at least 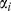 in 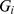, 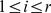 and the total number of intercluster edges 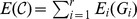 satisfies the following inequality 
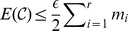
, where 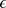 is a given parameter. However, we stress that JointCluster requires the number of clusters as input and relies on recursive bisection of the network. To overcome these drawbacks, JointCluster learns the parameters particular to a given data sets based on yet another cluster quality measure (therein, modularity).

Furthermore, our approach differs from *clustering of multislice, multiscale, or multilayer networks* [26], [27]. In this extension to the classical (single-layer) network concept, like in our setting, there are several networks over the same set of nodes; however, the corresponding nodes from two levels may be connected with inter-layer edges of prespecified weight. The influence of considering such edges in a generalization of the modularity yields a clustering which is conditioned on the structure of all networks, but also depends on the used weight of the included inter-layer edges. Moreover, approaches based on network alignment are not suitable for comparison of multiple networks over the same set of nodes, since the one-to-one correspondence between the nodes from different networks is given by definition.

### Special cases for COCONETS

We then have the following proposition based on Proposition 6.5 and Lemma 6.6 in [11]:

**Proposition 1**. *The optimal concurrent conditional clustering 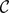 of 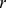 copies of* 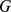, 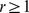 *consists of*:

1. *a single cluster, if 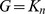, i.e., the complete graph on 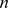 nodes*,

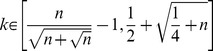
 *clusters of almost equal sizes, if 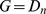, i.e., the cycle on 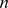 nodes*.

The following corollary shows that the optimal concurrent conditional clustering of 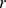 graphs of which 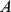 are complete graphs and 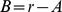 are cycles on 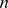 nodes, exhibits threshold property based on the value of 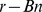. Therefore, the Corollary 2 establishes the existence of a threshold in the composition of the multiple networks at which switching appears between the 1-clustering and the finer partition containing clusters of almost equal sizes. This provides sound results for the intuitive understanding about the conditional aspects of the considered formulation of maximizing the sum of network modularities, whereby the clustering transforms as additional networks of particular structure are included.

**Corollary 2**. *Given 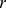 graphs of which 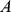 are complete graphs and 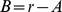 are cycles on the same set of 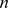 nodes, the optimal concurrent conditional clustering with minimum number of clusters 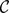 depends on the value of 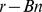 as follows:*

- *if* 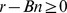, *then* 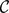 *is the clustering of optimal modularity for* 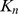,
- *if* 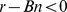, *then* 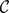 *has a factor of* 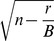 *more clusters than the clustering of optimal modularity for* 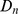.

*Proof.* First, we have the following observations for a clustering 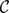 with 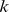 clusters: (1) if 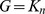, then 
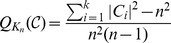
 and (2) if 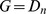, then 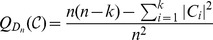. For a clustering of 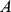 complete graphs and 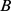 cycles on 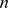 nodes, 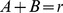, we then have: 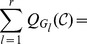
(2)

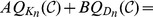
(3)

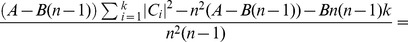
(4)

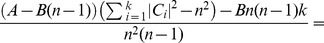
(5)

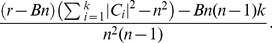
(6)


We note that for any 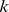, 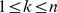, 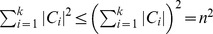, *i.e.*, 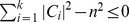. There are two cases to consider: (I) 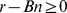 and (II) 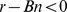.

Case (I): 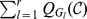 is maximized for 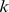 and clustering 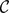 which simultaneously maximizes 
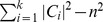
 and minimizes 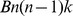. This is the case for 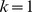, for which 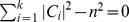 and, thus, 
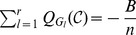
.

Case (II): For a fixed 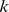, 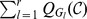 is maximized for clustering 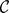 which minimizes 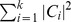. It is easy to show that 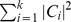 is minimized for clusters of almost equal sizes, so that 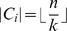, 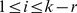, 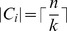, 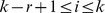, 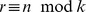. Treating the cluster sizes in 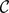 as real numbers, one obtains that 
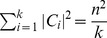
. Treating 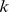 as a real number, and considering the continuous function 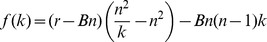, one obtains that 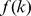 is maximized at 
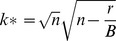
, since it is a fixed point for which the second derivative is negative (as 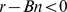). □ □

#### Extension of the greedy heuristic for modularity

Since COCONETS is NP-hard, here we propose a heuristic to find clusterings approximating the optimal, as defined in Definition 1. To this end, we need to define the score which will be used in combination with the clustering strategy. Since the greedy agglomerative heuristic for approximating the modularity of a single graph is a widely used solution [11], [28], here we propose and analyze the performance of an extension to the greedy agglomerative strategy for COCONETS.

Similarly to the case with a single network, the greedy heuristic starts with a clusterings of singletons in each of the 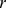 given networks. The extension is that it iteratively merges those two clusters that yield a clustering of the largest increase (the smallest decrease) in the *sum of modularities* over all networks (ties are arbitrarily broken). After 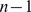 merges, the clustering that achieved the largest modularity over all networks and all merges is returned. More formally, this greedy heuristic uses a symmetric matrix 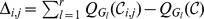, where 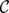 is the current clustering and 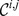 is obtained from 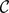 by merging clusters 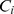 and 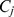 from 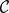. The rule for agglomeration then states that the clusters 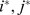 are merged if 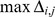 is achieved for the clusters 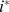 and 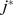. If there is more than one pair of clusters satisfying this condition, an arbitrary pair is selected.

## Applications

### Analysis of *E. coli* transcriptional response to stresses

The responses of *E. coli* to stress conditions have already been well investigated, resulting in characterization of the general and condition-specific components that regulate transcriptional changes underlying the adjustment to changing environments. Therefore, this setting represents an excellent test case for testing the power of the proposed approach. For networks extracted from transcriptomics data sets obtained under a given set of stresses, it would be expected that the clustering resulting from COCONETS captures adjustments of gene expression common to these stresses. Moreover, since the claims are based on clusterings obtained from networks modeling gene co-expression, COCONETS in fact facilitates quantification of the similarity of the concerted transcriptional response.

The time-resolved transcriptomics data set was obtained from [29], where changes in gene expression were monitored under four stress conditions, including: non-lethal temperature shifts, *i.e.*, heat and cold treatment, oxidative stress (by adding hydrogen peroxide), lactose-diauxic shift (*i.e.*, change of primary carbon source) and the entry in stationary phase (transition from late exponential growth to growth-arrest) relative to cultures grown under optimal conditions, referred to as control. The sampling was carried out from time points 10–50 min post-perturbation (at 10 min intervals) and two control time points before each perturbation for all conditions, except for stationary phase, where 60, 120, 145, 170 and 210 min post-perturbation were used for transcript profiling. Altogether, expression levels of 4,400 genes were monitored over seven time points in three replicates in each condition.

To identify the general and condition-specific responses, we first determined the differentially expressed genes for each of the conditions with respect to control. Differential expressed genes were identified by application of multiple moderated 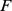-tests for two consecutive time-points, available in the limma R package, for each experimental condition separately on the 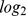-transformed expression levels using a hierarchical model [30]. Furthermore, differential expression of genes in stationary phase was derived using the grouping of time-points suggested in [29]. Finally, based on the obtained 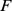-statistic (FDR-corrected 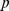-value 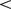 0.05), for each condition, genes were ranked and the top 122 genes (Table S1) which were annotated with either biological process (BP) or metabolic function (MF) terms from the GO ontology were selected. The latter ensures that the enrichment analysis is conducted only on well-characterized genes.

In total, for all five conditions, this resulted in 497 unique genes on which we then created five condition-specific relevance networks. For a given condition 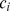, 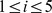, an edge is established between genes 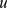 and 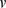 in the condition-specific network if their corresponding profiles (given by the means of the three replicates for each time point) are correlated above a statistically robust threshold 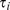 specific to condition 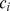. The condition-specific threshold 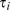 is determined to guarantee FDR of 0.05. The employed threshold values are: 0.98, 0.94, 0.95, 0.98, and 0.96 for stationary, heat, cold, oxidative stresses, and laxtose diauxie shift, respectively (Table 1). As a result, the condition-specific networks are all created on the same set of 497 nodes, but they contain 1415, 6750, 5563, 2199, and 5139 edges for stationary, heat, cold, oxidative stresses, and lactose diauxie, respectively. It is notable that although the network specific to heat stress is the densest (*i.e.*, with largest number of edges and average degree), it has a comparable average path length to that of the network specific to the stationary phase which is, incidentally, the sparsest. Moreover, while the networks from cold stress and lactose diauxie data are of comparable density, the former has the largest, while the latter the smallest diameter among the investigated networks (Table 1). In addition, the higher threshold values are not necessarily associated with smaller densities, since we found a weak correlation of −0.38 between the threshold and densities over the five networks. The discordance between the conclusions about similarities and differences between networks based on individual network properties led to the question of comparing the networks directly based on their edge sets.

**Table 1:** Condition-specific network properties and pairwise network similarities.

property	*c*	*h*	*ld*	*o*	*s*
**threshold**	0.95	0.94	0.96	0.98	0.98
**number of edges**	5563	6750	5139	2199	1415
**isolated nodes**	59	48	56	117	166
**average degree**	22.25	27.00	20.56	8.8	5.66
**maximum degree**	96	102	90	50	37
**average path length**	8.25	6.55	3.91	4.31	5.21
**diameter**	29	20	16	16	21
**transitivity**	0.71	0.72	0.62	0.58	0.58
***c***		0.83	0.12	0.07	0.14
***h***	0.83		0.13	0.07	0.13
***ld***	0.12	0.13		0.43	0.06
***o***	0.07	0.07	0.43		0.07
***s***	0.14	0.13	0.06	0.07	
**average Jaccard similarity**	0.29	0.29	0.18	0.16	0.10

The upper part of the table includes seven seminal network properties together with the thresholds used to establish the edges in the coexpression networks under five investigated stresses: cold (c), heat (h), lactose diauxie (ld), oxidative stress (o), and stationary phase (s). The lower part of the table includes the Jaccard similarity between the edge-sets of the condition-specific networks.

To this end, we first determined the Jaccard similarity between the edge sets for each pair of networks to identify which stresses induce the most divergent set of co-expressions (represented by the edges). It was shown that, on the level of metabolism, the stationary phase is the most divergent from the other investigated conditions [29]. Our results, Table 1, show that this is the case on the level of coexpression of the investigated genes, with an average Jaccard similarity of 0.10 for the stationary phase, followed by oxidative stress (0.16) and lactose diauxie (0.18). Considering all individual pairwise similarities of networks, cold and heat stresses are the closest, with Jaccard similarity of 0.83, followed by oxidative stress and lactose diauxie shift (0.43), Table 1. While these findings quantify the differences between networks, they do not reveal potential similarities with respect to finer sub-networks corresponding to network communities, which would support the similarities observed with respect to the other network properties examined above.

Next we apply the greedy heuristic for COCONETS with all five networks at once, each of the 10 pairs of networks as well as with the 5 individual networks. The similarity between the resulting 16 clusterings is examined by determining the adjusted Rand index, given in Table S2 (referred to in the following paragraphs). The most similar networks, with respect to the determined clusterings from individual stresses, are observed under heat and cold (0.83). The next most similar clusterings are observed for the lactose diauxie and oxidative stress experiments (0.44). The smallest average similarity is observed for the stationary condition, which implies that it is also deemed the most divergent with respect to the induced clustering. These observations are in line with the low Jaccard similarity of the respective pairs of networks based on their edge-sets.

It would be expected that the similar clusterings in the individual condition-specific networks would have comparable contributions to the CoCo clustering from the five condition-specific networks analyzed at once. This criterion is used as a validation for the greedy heuristic. The smallest contribution to the CoCo clustering over the five networks is indeed observed for the stationary phase (0.06), which was already determined to induce the most divergent transcriptional response. Moreover, the contributions of the pairs of cold and heat stress (0.32) as well as oxidative stress and lactose diauxie (0.39) are comparable (Table S2).

**Figure 3:**
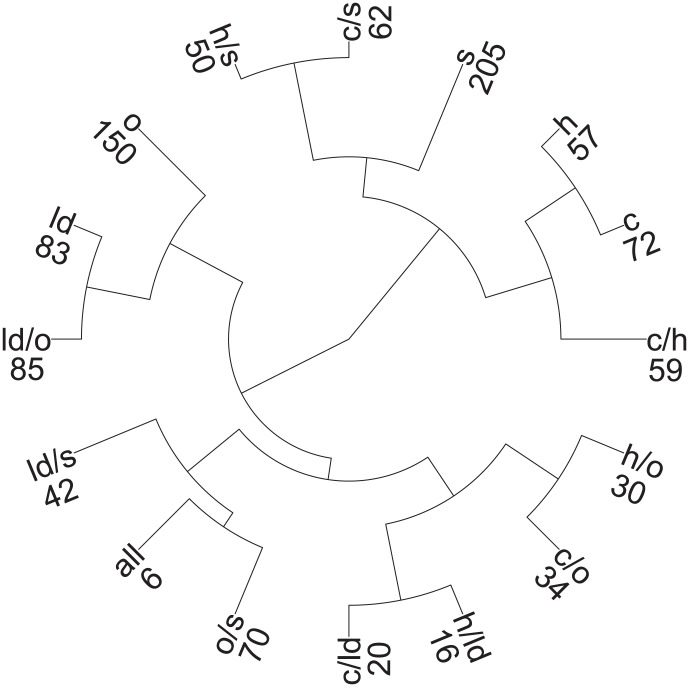
Clustering tree based on the adjusted Rand index values for the investigated CoCo clusterings. The clusterings from single networks are based on the greedy heuristic for approximating the MODULARITY problem. All other clusterings are based on the greedy heuristic for COCONETS. The tree is derived by agglomerative clustering with a distance matrix derived from the adjusted Rand index values for all pairwise comparisons of the obtained CoCo clusterings. The stress conditions are denoted as follows: cold (c), heat (h), lactose diauxie (ld), oxidative (o), and stationary phase (s); their pairwise combinations are marked with ‘/’, and the clustering over all five stresses, by ‘all’. The number of clusters in each CoCo clustering is included next to the abbreviations for the stresses.

Analogously, another criterion to validate the usage of the heuristic is the following: CoCo clusterings based on networks from a pair of conditions are expected to be the most similar to the clusterings induced by the individual conditions participating in the pair than to those induced by any other condition alone. As shown in Figure 3, this is indeed the case for all pairs of conditions. In addition, the CoCo clustering based on the pairs of networks from oxidative stress and stationary growth was the most similar to the CoCo clustering with all five networks (0.50), followed by heat stress and lactose diauxie (0.44), cold and oxidative stress (0.41), as well as lactose diauxie and stationary condition (0.39). Therefore, these three pairs of conditions have the largest influence on the CoCo clustering with all networks. Interestingly, while the similarity of the individual clustering from the stationary phase to the CoCo clustering with all five networks is the smallest (0.06), conditioned on the data from oxidative stress, it obtains the highest contribution (0.50) (see Table S2 for values).

By investigating the combination of pairs of networks, we can further confirm the validity of the approach: The largest similarity between CoCo clusterings for two pairs of networks, from conditions 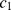, 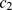 and 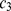, 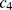, with 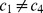, is expected when 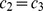, *i.e.*, when one of the conditions is shared. Moreover, the ordering of the similarities between 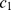, 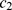 and 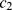, 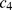 follows the ordering of the similarities between the clusterings for the individual conditions 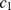 and 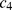. For instance, when the condition 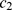 is lactose diauxie, the largest similarity is indeed determined for CoCo clusterings from lactose diauxie, cold stress and lactose diauxie, heat stress, as the clusterings from heat and cold stress are the most similar; the same holds if the condition 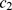 is oxidative stress and the stationary phase (Table S2).

While these findings illustrate the structure-driven validity of the obtained network clusterings, they do not on their own provide biological insights apart from the similarity between stress conditions. To obtain biological insights, we determine the GO terms (MF and BP) for which the determined network communities are enriched. It is expected that with increasing number of networks, the enriched MF and BP terms in the corresponding CoCo clustering would correspond to molecular functions and biological processes preserved in a more general response corresponding to the larger number of conditions. With the five networks, the CoCo clustering contains six clusters of which three are enriched for MF and BP terms at significance level of 0.05: (i) metabolism of sulfur amino acids, pyrimidine nucleotides, and amines as well as sulfur utilization, (ii) oxidoreduction-driven active transmembrane transporter activity and macromolecule and protein metabolic processes, and (iii) structural constituent of ribosome, (r)RNA binding, and translation.

Analysis of our data sets for transcripts indicative for anaerobiosis demonstrated the absence of any oxygen shortage under optimal growth conditions. In contrast, there was a slight induction of genes associated with aerobic respiration, *e.g.*, ubiquinone oxidoreductase (nuoH, nuoL, nuoN), generating transmembrane proton gradient. Induction of expression of genes associated with hypoxia was, however, observed after lactose-diauxie shift, oxidative stress, and more pronounced during heat and stationary phase [29]. Other biological processes that depend on proton gradient are ATP synthesis and transmembrane transport. However, in contrast to genes involved in ATP synthesis, which decrease after all perturbations, genes encoding general transport increase during lactose diauxic shift and oxidative stress [31]. Therefore, the clustering results are also in line with the observed similarity between lactose diauxic shift and oxidative stress (Table 1). In addition, general stress responses aim at reducing energy expenditure through repression of genes involved in growth, cell division, and protein synthesis [32]. It has already been shown that the stringent response involves the down-regulation of genes involved in transcription and translation [33]. Altogether, these support the results from the enrichment analysis on the CoCo clustering over five networks.

The most similar CoCo clustering with pairs of conditions results from the networks under oxidative stress and stationary phase (0.50) (Figure 3). In addition to the already mentioned GO terms enriched in the clustering over all networks, the following terms are specifically enriched under oxidative stress and stationary phase: (i) post-translational protein modifications (phosphorylation), chemotaxis, cell communication, signal transduction, two-component sensor activity and protein kinase activity, and (ii) disaccharide (lactose) metabolic processes. The first group of enriched terms corresponds to the most prominent effects of oxidative stress, affecting protein kinases and signaling. The second group matches well the fact that *E. coli* preferably metabolizes glucose, while other sugars, such as lactose, are only consumed after depletion of glucose resulting in temporary growth arrest.

Therefore, the findings from the comparison of clusterings, extracted from the networks by combining 497 genes which are not differentially expressed over all stresses, demonstrate that COCONETS can identify both the general as well as specific responses. Most notably, this was not achieved by investigating the intersection of differentially expressed genes in a given subset of stresses, but rather by joined investigation of condition-specific networks with respect to shared community structure maximizing the sum of modularities over all networks.

### Analysis of *Arabiodpsis thaliana* metabolic response to changing environmental conditions

In addition, we applied COCONETS to a publically available metabolomics data set from *Arabidopsis thaliana* plants exposed to eight environmental conditions differing in light intensity and/or temperature [34]. Six-week-old *Arabidopsis thaliana* plants, grown in soil at 21° C and 150
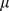
E m^−2^ sec^−1^ (control), were either kept in the control condition or were transferred to seven different environments reflecting a light gradient ranging from darkness to high-light stress and a temperature gradient from 4° C over 21° C to 32° C. For each condition 23 time points (including the zero time point, before stress application) are taken to follow the stress response, ranging from 5 to 1280 min after exposure to the different conditions. To identify similarities and differences between these conditions, either one (light intensity or temperature) or both environmental parameters were changed. This resulted in the following eight environmental conditions: (i) 4° C and darkness (abbreviated as 4-D), (ii) 21° C and darkness (21-D), (iii) 32° C and darkness (32-D), (iv) 4° C and 85
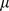
E m^−2^ sec^−1^(light; 4-L), (v) 21° C and 75
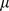
E m^−2^ sec^−1^ (low light; 21-LL), (vi) 21° C and 300
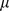
E m^−2^ sec^−1^ (high light; 21-HL), (vii) 32° C and 150
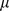
E m^−2^ sec^−1^ (light; 32-L), and plants kept at the original conditions (21° C and 150
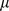
E; 21-L). Metabolites were measured in six replicates from single Arabidopsis rosettes. In total, metabolite levels are available for 101 metabolites across conditions and time points.

For each condition we then created a relevance network based on the Pearson correlation coefficients between the time-resolved metabolite levels by using the median of the six biological replicates for each metabolite [35]. An edge between metabolites is established in a similar way as for the gene-expression network by only considering edges which pass a statistically robust threshold 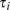 specific to an individual condition 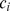. The condition-specific threshold 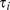 is determined to guarantee FDR of 0.01. The employed threshold values are: 0.665, 0.630, 0.625, 0.670, 0.635, 0.615, 0.650, and 0.640 for 21-L, 4-D, 21-D, 32-D, 4-L, 21-LL, 21-HL, and 32-L, respectively (Table 2). The condition-specific networks all contain the same set of nodes corresponding to the 101 metabolites but show a varying number of edges connecting them and range from 132 for 21-L to 502 for 32-D (see Table 2 for other network properties).

**Table 2:** Condition-specific network properties and pairwise network similarities for eight environmental conditions in *Arabidopsis thaliana*.

property	*21-L*	*21-D*	*4-L*	*4-D*	*32-L*	*32-D*	*21-LL*	*21-HL*
**threshold**	0.665	0.63	0.625	0.67	0.635	0.615	0.65	0.64
**number of edges**	132	393	409	166	372	502	227	296
**isolated nodes**	33	15	11	26	16	18	23	17
**average degree**	2.61	7.78	8.10	3.29	7.37	9.94	4.50	5.86
**maximum degree**	9	22	19	12	20	27	18	19
**average path length**	3.25	2.41	4.48	2.60	4.10	1.83	3.27	2.78
**diameter**	8	6	12	8	12	6	11	8
**transitivity**	0.56	0.65	0.68	0.61	0.71	0.74	0.62	0.62
***21-L***		0.15	0.17	0.27	0.19	0.12	0.28	0.21
***21-D***	0.15		0.16	0.21	0.26	0.40	0.30	0.20
***4-L***	0.17	0.16		0.16	0.20	0.16	0.16	0.28
***4-D***	0.27	0.21	0.16		0.19	0.14	0.23	0.17
***32-L***	0.19	0.26	0.20	0.19		0.26	0.24	0.23
***32-D***	0.12	0.40	0.16	0.14	0.26		0.20	0.15
***21-LL***	0.28	0.30	0.16	0.23	0.24	0.20		0.20
***21-HL***	0.21	0.20	0.28	0.17	0.23	0.15	0.20	
**average Jaccard similarity**	0.20	0.26	0.19	0.18	0.23	0.22	0.22	0.21

The upper part of the table includes seven seminal network properties together with the thresholds used to establish the edges in the correlation networks of metabolites under eight investigated stresses: 4 C and darkness (4-D), 21 C and darkness (21-D), 32 C and darkness (32-D), 4 C and light (4-L), 21 C and low-light (21-LL), 21 C and high light (21-HL), and 32 C and light (32-L). The lower part of the table includes the Jaccard similarity between the edge-sets of the condition-specific networks.

**Figure 4:**
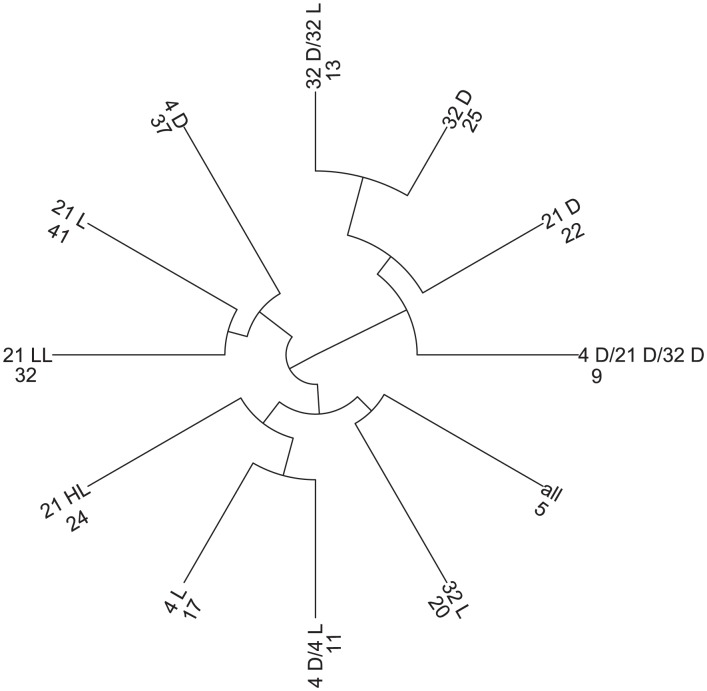
Clustering tree based on the adjusted Rand index values for the investigated CoCo clusterings. The clusterings from single networks are based on the greedy heuristic for approximating the MODULARITY problem. All other clusterings are based on the greedy heuristic for COCONETS. The tree is derived by agglomerative clustering with a distance matrix derived from the adjusted Rand index values for all pairwise comparisons of the obtained CoCo clusterings. The stress conditions are denoted as follows: 4° C and darkness (4 D), 21° C and darkness (21 D), 32° C and darkness (32 D), 4° C and light (4 L), 21° C and low-light (21 LL), 21° C and high light (21 HL), 32° C and light (32 L), and 21° C and light (21 L, control); their pairwise combinations are marked with ‘/’, and the clustering over all five stresses, by ‘all’. The number of clusters in each CoCo clustering is included next to the abbreviations for the stresses.

By determining the Jaccard similarity between the edge-sets of the condition-specific network (Table 2) we observe that the network at 4-D differs the most from the other networks (average Jaccard similarity 0.18), followed by that at 4-L (Jaccard similarity of 0.19); moreover, the networks from 21-D and 32-D are closest to each other (Jaccard similarity 0.40). To analyze potential similarities in network communities we applied the greedy heuristic for COCONETS with each condition-specific separately as well as all eight networks at once. Furthermore, we investigated a combination of networks from conditions with a temperature of 4° C (4-L and 4-D) and 32° C (32-L and 32-D) as well as for darkness treatment (4-D, 21-D, and 32-D). In total, 12 clusterings are obtained which are examined by the pairwise adjusted Rand index of the clusterings (Table S3, Figure 4). The most similar clusterings for individual environmental conditions are observed for 21-D and 32-D (0.58) indicating a similar response to the different treatments which was already shown in the analysis of Caldana *et al.* [34]. They also noted that the third darkness condition with the temperature kept at 4° C (4-D) only showed a small overlap with the 21-D and 32-D responses, which is further supported by the similarities of condition-specific clusterings as well as between individual darkness conditions and the overall clustering (21-D/4-D/32-D) (Table S3, Figure 4). Based on all 12 clusterings the highest similarity (0.73) is observed between 32-D and combinations of 32° C conditions (32-L/32-D) indicating that the clustering of 32-D highly represents the general effect of high temperature. The CoCo clustering from the eight condition-specific networks analyzed at once has the highest similarity to 32-L (0.31), but generally a low similarity to the clusterings of individual networks. The low similarity may largely be due to the high range of different simultaneosly investigated conditions. Therefore, the findings from the CoCo clustering of metabolite-correlation networks show another example of possible application for the proposed approach to get further insights not only from condition-specific clusterings but also clustering of different conditions at once highlighting the response similarities.

## Conclusions

Based on the analysis of coexpression and metabolic correlation networks extracted from transcriptomics and metabolomics data obtained from multi-stress experiments with *E. coli* and *A. thaliana*, respectively, we demonstrated that the proposed approach COCONETS can be used to dissect the subtle similarities and differences between conditions. In addition, our findings indicated that in combination with enrichment analysis, COCONETS offers a novel means to identify molecular processes underpinning the general as well as condition-specific responses of different levels of biological organization in various organisms.

## Supporting Information

Table S1 **Considered differentially-expressed genes for each condition.**
(XLSX)

Table S2 **Adjusted Rand index values for the investigated CoCo clusterings of the co-expression networks of different stresses in** ***E.coli*** **.**
(XLSX)

Table S3 **Adjusted Rand index values for the investigated CoCo clusterings of metabolite correlation networks from different environmental conditions of** ***Arabidopsis thaliana*** **.**
(XLSX)
